# Integrative Multi‐Omics and Experimental Validation Define RELB as an Immune‐Related Prognostic Driver in Ovarian Cancer

**DOI:** 10.1111/cbdd.70409

**Published:** 2026-10-13

**Authors:** Wenshu Leng, Xingyue Fan, Yichen Fang

**Affiliations:** ^1^ Department of Gynecologic Oncology Jiangsu Cancer Hospital, Jiangsu Institute of Cancer Research, The Affiliated Cancer Hospital of Nanjing Medical University Nanjing China; ^2^ Department of Rehabilitation The Fourth People's Hospital of Lianyungang, Affiliated to Kangda College of Nanjing Medical University Lianyungang China

**Keywords:** drug sensitivity, immune microenvironment, key genes, metabolic reprogramming, molecular docking, multi‐omic, ovarian cancer

## Abstract

Ovarian cancer (OC) is one of the most aggressive gynecological malignancies worldwide. The tumor microenvironment and transcriptomic alterations play important roles in OC progression and therapeutic response. Integrative bulk and single‐cell transcriptomic analyses may facilitate the identification of prognostic biomarkers and potential therapeutic targets. Multi‐Omic datasets from TCGA and GEO were analyzed. Immune‐related genes were first integrated with survival information and screened using a random survival forest model to identify five core genes. GO/KEGG analyses were performed on differentially expressed genes, while GSEA and GSVA were used to characterize pathways associated with the core genes. Single‐cell RNA‐seq data were processed to determine cell‐type‐specific expression patterns. Molecular docking predicted potential gene‐drug interactions. CCK‐8, Transwell, and flow cytometry assays assessed the functional role of RELB in OC cells. Five core genes‐RELB, CXCL9, PI3, TRAC, and CXCL11‐were identified through integration of immune‐related genes and random survival forest analysis. Drug sensitivity analysis showed that the expression of all five genes was associated with sensitivity to KU‐55933. High expression of key genes was associated with altered immune‐related features, while CXCL9, CXCL11, and TRAC were positively associated with CD8+ T‐cell abundance and cytolytic activity. Single‐cell sequencing identified seven cell types, among which CXCL9 and CXCL11 were highly expressed in monocytes, TRAC in T cells, and PI3 in epithelial cells. Molecular docking predicted a potential interaction between PI3 and 6atu‐PROGESTERONE, with a binding energy of −6.35 kcal/mol. RELB was highly expressed in OC cells, and its knockdown significantly inhibited cell proliferation, migration, and invasion while promoting apoptosis. This study identifies a five‐gene prognostic signature linked to the immune microenvironment in ovarian cancer. Among them, RELB showed a functional role in OC cell malignant phenotypes, supporting its potential as a biomarker and therapeutic target. Computational drug sensitivity and docking analyses provide hypothesis‐generating evidence for potential therapeutic associations that require experimental validation.

## Graphical abstract

Integrated multi‐omics identified key genes associated with the immune microenvironment in ovarian cancer, and functional assays validated the role of key genes in ovarian cancer.

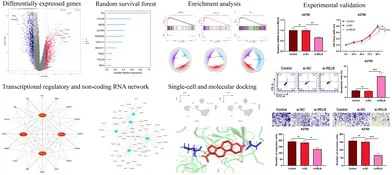

## 1 Introduction

Ovarian cancer (OC) ranks among the most lethal and progressive gynecological malignancies across the globe. Each year, it accounts for more than 314,000 newly diagnosed cases and around 213,000 fatal outcomes worldwide; epithelial ovarian cancer (EOC) constitutes over 90% of all OC diagnoses (Wang, Zhu, et al. 2025). This type of OC originates in the surface lining of the ovaries or the inner lining of the fallopian tubes (Bryant et al. 2022). High‐grade serous ovarian cancer (HGSOC) ranks as the predominant histological variant among EOC subtypes. It is characterized by the presence of TP53 somatic mutations in nearly all cases and homologous recombination deficiency (HRD) occurs in nearly half of all clinical cases, encompassing genetic aberrations affecting BRCA1 alongside BRCA2 (Jitmana et al. 2024). Current evidence and guidelines recommend testing all patients with EOC for germline pathogenic mutations in BRCA1 and BRCA2 and other OC susceptibility genes. This not only guides treatment decisions, such as the appropriateness of maintenance therapy with poly (ADP‐ribose) polymerase inhibitors (PARPi), but also enables more personalized treatment strategies (Gaillard et al. 2025). Despite advances in surgical cytoreduction, platinum‐based chemotherapy, PARPis, anti‐angiogenic agents, and antibody–drug conjugates, many patients ultimately develop recurrent or platinum‐resistant disease, and long‐term survival remains unsatisfactory (Bryant et al. 2022; Colombo and Ledermann 2021; Li et al. 2024; Moore et al. 2023). These clinical challenges highlight the need for improved molecular stratification and the identification of biomarkers that can predict prognosis and reveal actionable therapeutic vulnerabilities.

While immune checkpoint inhibitors (CPI) yield potent therapeutic benefits across many solid malignancies, their standalone clinical value against EOC is limited. Current research has shifted its focus to the combination of CPI with other emerging treatment modalities (Bogani et al. 2025). A growing body of evidence suggests that, apart from cancerous cellular components, cells within the tumor microenvironment (TIME) also profoundly influence tumor biology and clinical characteristics (Chang et al. 2024). Similarly, the tumor immune microenvironment (TIME) significantly influences treatment response and prognosis in patients receiving immunotherapy (Liu et al. 2022). Therefore, systematic identification of immune‐associated molecular regulators and their cell‐specific expression patterns is warranted to clarify their contribution to OC progression and treatment response. In particular, investigating key immune‐related genes, including chemokine‐associated factors, T‐cell‐related molecules, and transcriptional regulators, may facilitate the identification of prognostic biomarkers and potential therapeutic targets for OC.

OC is also characterized by extensive transcriptomic alterations that contribute to tumor heterogeneity and altered communication between malignant and immune cells (Yi et al. 2023). However, conventional bulk transcriptomic analyses measure the average expression of genes across heterogeneous tumor tissues and therefore cannot adequately determine the cellular sources or cell‐type‐specific functions of candidate genes. Single‐cell RNA sequencing (scRNA‐seq) overcomes this limitation by analyzing the transcriptional program at the single‐cell level, enabling the characterization of the heterogeneity of tumor and immune cell populations (Sun et al. 2025). Therefore, integrating bulk and single‐cell transcriptomic data helps to first identify molecular candidates with prognostic relevance at the patient level and then determine their distribution in cells and their potential involvement in the TIME. This integrated strategy is expected to improve the biological interpretation of prognostic biomarkers and facilitate the discovery of molecular targets with potential therapeutic significance.

The purpose of this study is to systematically identify immune‐related genes closely related to the prognosis of OC and to elucidate their biological and cytological significance in the tumor microenvironment. Although multi‐omic approaches hold promise for refining personalized cancer therapy, their clinical utility in OC remains to be established. Our study contributes to this effort by prioritizing candidate biomarkers and functional drivers for further translational investigation.

## 2 Materials and Methods

### 2.1 Data Acquisition

Single‐cell RNA sequencing datasets, including GSE184880 and 11 additional transcriptomic profiles (totaling 12 datasets with 5 control and 7 malignant samples), were obtained from the Gene Expression Omnibus (GEO; https://www.ncbi.nlm.nih.gov/geo) database. This dataset provides comprehensive single‐cell phenotypic characteristics of human high‐grade serous ovarian cancer (HGSOC), including 59,324 cells (Xu et al. 2022). It has been widely used as a representative single‐cell reference sample in multiple independent ovarian cancer studies (Di et al. 2025; Meng and Sun 2024).

Bulk transcriptomic data, comprising 422 ovarian tumor samples from The Cancer Genome Atlas (TCGA, https://www.cancer.gov/tcga) and 88 normal ovarian tissue samples from GTEx (Genotype‐Tissue Expression, https://gtexportal.org), were downloaded and merged. After removing batch effects via ComBat normalization, the merged bulk dataset was used to perform differential expression analysis, functional enrichment analyses (including GO/KEGG, GSEA, and GSVA), and random survival forest‐based prognostic gene screening. Immune infiltration correlations and drug sensitivity predictions were subsequently evaluated based on the identified gene signatures. Download the immune‐related genes from the website immport database (https://www.immport.org/) for subsequent Veen analysis.

### 2.2 Differential Analysis

The Limma R toolkit was utilized to conduct gene differential expression profiling. We explored the distinct molecular regulatory patterns underlying tumor pathogenesis and screened divergent transcripts between nontumor and malignant tissues. Candidate genes were filtered following strict thresholds of *p* value < 0.05 and |logFC| > 2; meanwhile, visualizations including volcano maps and heat diagrams were constructed to illustrate the overall distribution of these altered transcripts.

### 2.3 Functional Enrichment Analysis

To identify the biological roles and signal cascades linked to this gene collection, we adopted the R package “Cluster Profiler” for genetic annotation and systematic dissection of relevant functional interactions. Functional enrichment patterns were assessed via Gene Ontology (GO) and Kyoto Encyclopedia of Genes and Genomes (KEGG) annotations. Any GO terms and KEGG enrichment entries with *p* values and *q*‐values less than 0.05 were considered significant categories.

### 2.4 Random Survival Forest Analysis

To perform dimensionality reduction and preselect prognostic features, a random survival forest (RSF) model was constructed using the randomForestSRC R package, with overall survival time and survival status as outcome variables and the expression levels of candidate target genes (the overlapping set of differentially expressed genes and immune‐related genes) as predictor variables. The model was built with 500 survival trees (ntree = 500) and a fixed random seed (seed = 1080) to ensure reproducibility. Model stability was monitored via the out‐of‐bag (OOB) error rate as the number of trees increased. Variable relative importance (VRI) scores were then extracted from the fitted model, and genes with importance values exceeding a predefined threshold (imp.cutoff = 0.1) were retained as candidate prognostic genes. Subsequently, Kaplan–Meier survival analysis with log‐rank tests was performed on these selected genes to evaluate their associations with patient prognosis, and genes with *p* < 0.05 were considered statistically significant and designated as the key genes for further investigation.

### 2.5 Drug Sensitivity Analysis

Drug sensitivity was predicted using the oncoPredict R package based on the Genomics of Drug Sensitivity in Cancer (GDSC) database (https://www.cancerrxgene.org/). IC_50_ values for each sample were estimated via ridge regression, with key parameters configured as follows: batch Correct = “eb” for empirical Bayes batch correction (ComBat algorithm), power Transform Phenotype = TRUE for phenotype transformation, remove Low Varying Genes = 0.2 to exclude 20% of low‐variation genes, and minNumSamples = 10 as the minimum sample size for drugs in the training set. Model prediction accuracy was evaluated using 10‐fold cross‐validation, while all other parameters were kept at default settings.

### 2.6 Immune Infiltration and Immune‐Related Correlation Analysis

Immune infiltration was evaluated using single‐sample gene set enrichment analysis (ssGSEA) performed with the GSVA R package (gsva function, method = “ssgsea”, kcdf = “Gaussian”, abs.ranking = TRUE) on the normalized transcriptomic expression matrix. This approach generated enrichment scores for 29 immune cell populations, reflecting their relative infiltration levels in the tumor microenvironment.

Additionally, ssGSEA was used to score multiple immune‐related functional signatures and pathways, including antigen‐presenting cell (APC) co‐inhibition, APC co‐stimulation, cytolytic activity, chemokine receptor (CCR) activity, T‐cell co‐stimulation, and pro‐inflammatory status. Samples were stratified into high‐ and low‐expression groups based on the expression level of each key gene, and differences in immune infiltration scores and functional scores between the two groups were assessed using Wilcoxon rank‐sum tests. Spearman correlation analyses were performed to examine the relationships between key gene expression and immune cell populations, immune‐related functional signatures, and immunoregulatory pathways, with particular focus on tumor‐infiltrating lymphocytes (TILs), Th1 cells, dendritic cells (DCs), macrophages, T‐cell co‐stimulation, and APC co‐inhibition. To further explore associations with a broader spectrum of immune‐related factors, the Tumor‐Immune System Interactions Database (TISIDB) was utilized to systematically evaluate correlations between key genes and immunomodulators, chemokines, and chemokine receptors.

### 2.7 Gene Set Enrichment Analysis (GSEA)

According to the transcriptional abundance of key genes in tested specimens, all specimens were stratified into high‐ and low‐expression subgroups. GSEA was then performed to identify pathway discrepancies between the two subgroups using the annotated gene sets (version 7.0) retrieved from the Molecular Signature Database (MSigDB). Significantly enriched gene sets were ranked by consistency scores, with an adjusted *p* value < 0.05 considered statistically significant.

### 2.8 Gene Set Variation Analysis (GSVA)

Gene set collections were acquired from MSigDB (v7.0). GSVA was then applied to generate enrichment scores for each gene set per specimen, and the resulting scores were used to assess biological function differences across samples.

### 2.9 Transcription Factor Regulatory Network

The transcription factor (TF) regulatory relationships of the five key genes were investigated using the RcisTarget R package. The gene set containing the key genes was used as the input, and RcisTarget was applied to identify TFs whose DNA‐binding motifs were significantly enriched in the regulatory regions of the target genes. Motif enrichment was evaluated using the normalized enrichment score (NES), and TF–target gene pairs meeting the predefined significance threshold were retained for subsequent network construction. The resulting TF–key gene regulatory relationships were visualized using Cytoscape, with TFs and key genes represented as nodes and their predicted regulatory relationships represented as edges.

### 2.10 Noncoding RNA Network Associated With Key Genes

Potential miRNA regulators of the five key genes were identified using the miRcode database. The five key genes were queried individually, and miRNA–mRNA interactions supported by the database were retrieved. The resulting miRNA–key gene interaction pairs were integrated to construct a regulatory network, which was subsequently visualized using Cytoscape.

### 2.11 Single Cell Data Quality Control

Single‐cell expression matrices were imported and processed using the Seurat package. Cells were first filtered by requiring at least 200 detected genes (min.features = 200), and quality‐control metrics including nCount_RNA (total UMI counts), nFeature_RNA (number of detected genes), percent.mt (mitochondrial gene proportion), and percent.ribo (ribosomal gene proportion) were assessed. For these metrics, outliers were identified using the median absolute deviation (MAD) method, and cells with values exceeding 3 MADs from the median were excluded. Subsequently, potential doublets were identified and removed from each sample individually using DoubletFinder v2.0.4. The remaining cells were used for downstream normalization, dimensionality reduction, clustering, and cell‐type annotation.

### 2.12 Dimension Reduction and Clustering of Single‐Cell Data

After quality control, single‐cell gene expression data were normalized using the Log Normalize method in Seurat with a scaling factor of 50. Cell‐cycle scores were calculated using Cell Cycle Scoring, and highly variable genes were identified using Find Variable Features. The normalized data were scaled using ScaleData, with mitochondrial and ribosomal gene expression proportions regressed out. Principal component analysis was performed using RunPCA, and informative principal components were selected for subsequent analysis. Harmony was used to correct batch effects, followed by RunUMAP for dimensionality reduction and visualization. Cell clustering was subsequently performed using the Harmony‐corrected principal components.

### 2.13 Cell Annotation

Cell types were annotated using canonical marker genes curated from CellMarker, PanglaoDB, and published studies, with SingleR used as a supplementary automated annotation tool. Cluster‐specific marker genes were identified using FindAllMarkers with only.pos = TRUE, min.pct = 0.25, and logfc.threshold = 0.25. Cell identities were assigned by integrating marker‐gene expression and SingleR annotation results.

### 2.14 Molecular Docking

With reference to UniProt accession numbers and sequence homology of key genes, the three‐dimensional (3D) structural information of their encoded proteins was acquired from the Protein Data Bank (PDB; https://www.rcsb.org/). Potential compound‐gene associations of key genetic targets were predicted via the DGldb (Drug‐Gene Interaction Database, https://www.dgidb.org/) to identify relevant key compounds. We subsequently acquired the molecular configurations of these compounds from the PubChem (Public Chemistry Database, https://pubchem.ncbi.nlm.nih.gov/) to acquire detailed structural characteristics of the active pharmaceutical ingredients. Molecular docking analysis was carried out via AutoDuck 4.2. Protein structures were prepared by removing unnecessary water molecules and adding polar hydrogen atoms and partial atomic charges, whereas ligand structures were converted into the required docking format after structural optimization and assignment of atomic charges. A grid‐based docking procedure was used to define the search space around the predicted or reported ligand‐binding region. The grid spacing and the grid‐box center and dimensions were specified according to the corresponding protein–ligand binding site. A genetic algorithm was adopted as the primary docking strategy; the default parameters of AutoGrid were used. For each pair of protein‐ligand, 50 independent docking simulations were conducted. The docking model exhibiting the most favorable binding affinity was chosen for subsequent presentation, and all output data were loaded into PYMOL for graphical visualization, enabling the identification of interaction sites between target proteins and small molecule compounds.

### 2.15 Cell Culture and Transfection

The human ovarian cancer cell lines SKOV3 and A2780, as well as the human normal ovarian epithelial cell line HOSEpiC, were all obtained from the American Type Culture Collection (ATCC, VA, USA). SKOV3 cells were cultured in McCoy's 5A medium (Gibco) containing 10% fetal bovine serum (FBS, Gibco) and 1% penicillin–streptomycin (P/S, Gibco). A2780 cells were cultured in RPMI‐1640 medium (Gibco) containing 10% FBS and 1% P/S. HOSEpiC cells were cultured in RPMI‐1640 medium containing 10% FBS and 1% P/S. All cells were cultured in a humidified incubator at 37°C with 5% CO_2_.

A2780 cells were seeded at an appropriate density in 6‐well plates and transfected when the cell confluence reached 60%–70%. The si‐RELB and negative control si‐NC were synthesized by GenePharma (Shanghai, China). After transfection using Lipofectamine 3000 transfection reagent (Invitrogen, CA, USA), the cells were collected at 48 h.

### 2.16 Cell Counting Kit‐8 (CCK‐8) Assay

A2780 cells were seeded at a density of 3 × 10^3^ cells per well in a 96‐well plate and incubated in a 37°C, 5% CO_2_ incubator overnight. After the cells adhered to the plate, siRNA transfection was performed. The experiment was divided into three groups: the untreated control group (Control), the negative control transfection group (si‐NC), and the RELB knockdown group (si‐RELB). Five replicate wells were set for each group. Detection was conducted at 0 h, 24 h, 48 h, 72 h, and 96 h after transfection. At each time point, 10 μL of CCK‐8 reagent (Beyotime, Nanjing, China) was added to each well and incubated at 37°C in the dark for 2 h. The optical density (OD) value of each well was measured at 450 nm using a microplate reader. All experiments were independently repeated three times.

### 2.17 Cell Apoptosis Experiment

Cell apoptosis was determined by flow cytometry. In brief, the Control, si‐NC, and si‐RELB groups were set up. After treatment, the cells were collected, washed with cold PBS, and incubated at 4°C in the dark for 30 min with Annexin V‐FITC (BioTECH, Nanjing, China) for labeling. Then, propidium iodide was added for incubation. The analysis was performed using a flow cytometer and combined with FlowJo software (BD Biosciences, USA).

### 2.18 Cell Migration and Invasion Experiment

The migration and invasion abilities of the cells were evaluated using Transwell culture dishes (8 μm pore size; Corning). The cell invasion experiment was conducted using a culture plate coated with Matrigel (Corning, NY, USA). 200 μL of A2780 cell suspension was added to the upper chamber of the culture dish, and 600 μL of RPMI‐1640 medium containing 10% FBS was added to the lower chamber as a chemoattractant. After 48 h, the cells that did not penetrate the membrane at the bottom of the upper chamber were gently wiped off with a cotton swab. The cells that penetrated the membrane were fixed in 4% paraformaldehyde, stained with 0.1% crystal violet, and counted.

### 2.19 qRT‐PCR Assay

According to the manufacturer's instructions, total RNA was extracted using TRIzol reagent (Invitrogen, Carlsbad, CA, USA). 1 μg of total RNA was reverse‐transcribed into cDNA using PrimeScript RT Master Mix (Takara Bio, Kusatsu, Japan). Subsequently, qPCR amplification was performed using SYBR Premix Ex Taq II (Takara Bio, Kusatsu, Japan) on the LightCycler 480 real‐time fluorescence quantitative PCR system (Roche, Basel, Switzerland). GAPDH was used as the internal reference gene, and the relative expression level of the target gene was calculated using the 2^−ΔΔCt^ method. The primer sequences were: RELB, Forward, 5′‐TCGACGAGTACATCAAGGAGA‐3′, Reverse, 5′‐CAGGGTGACCGTGCTCAG‐3′; PI3, Forward, 5′‐TGTTGAATCCCCCTAACCGC‐3′, Reverse, 5′‐TCACTGGGGAACGAAACAGG‐3′; TRAC, Forward, 5′‐ACCAACTGGATCCTACCCGA‐3′, Reverse, 5′‐ATGTCAGGCAGTGACAAGCA‐3′; GAPDH, Forward, 5′‐GTCTCCTCTGACTTCAACAGCG‐3′, Reverse, 5′‐ACCACCCTGTTGCTGTAGCCAA‐3′.

### 2.20 Statistical Analysis

The R language (version 4.3.0) was used for all statistical analyses, and a *p* value of less than 0.05 was regarded as statistically significant. All experimental data are presented as mean ± standard deviation and analyzed using GraphPad Prism 8.0 (GraphPad Software Inc., USA). The student's *t*‐test was used for comparisons between two groups, and one‐way ANOVA followed by Tukey's post hoc test was employed for comparisons among multiple groups. A difference was considered statistically significant if *p* < 0.05.

## 3 Results

### 3.1 Differential Gene Expression Profiling and Enrichment Analysis

The limma package was applied to screen differentially expressed genes between healthy controls and disease cohorts. Differential genes were screened under the threshold of ∣LogFC∣ > 2 and a *p* value below 0.05. In this study, 2500 aberrantly expressed genetic transcripts were screened out, consisting of 1065 genes with increased expression levels and 1435 ones with reduced transcription abundance. Further visualization analyses were performed to construct volcano maps and expression heatmaps for those aberrantly transcribed genes (Figure 1A,B). Next, functional enrichment profiling was conducted for all dysregulated genes. GO enrichment outcomes revealed that these aberrantly transcribed genes mainly clustered within biological processes linked to chromosome segregation, a process central to the chromosomal instability characteristic of HGSOC (Tamura et al. 2020); and cell–cell junction organization, which is critically involved in OC metastasis and genomic integrity (Villagomez et al. 2025) (Figure 1C); KEGG results indicated that the differentially expressed genes were primarily enriched in pathways such as Ras signaling pathway, a well‐established oncogenic driver in OC (Therachiyil et al. 2022) (Figure 1D).

**FIGURE 1:**
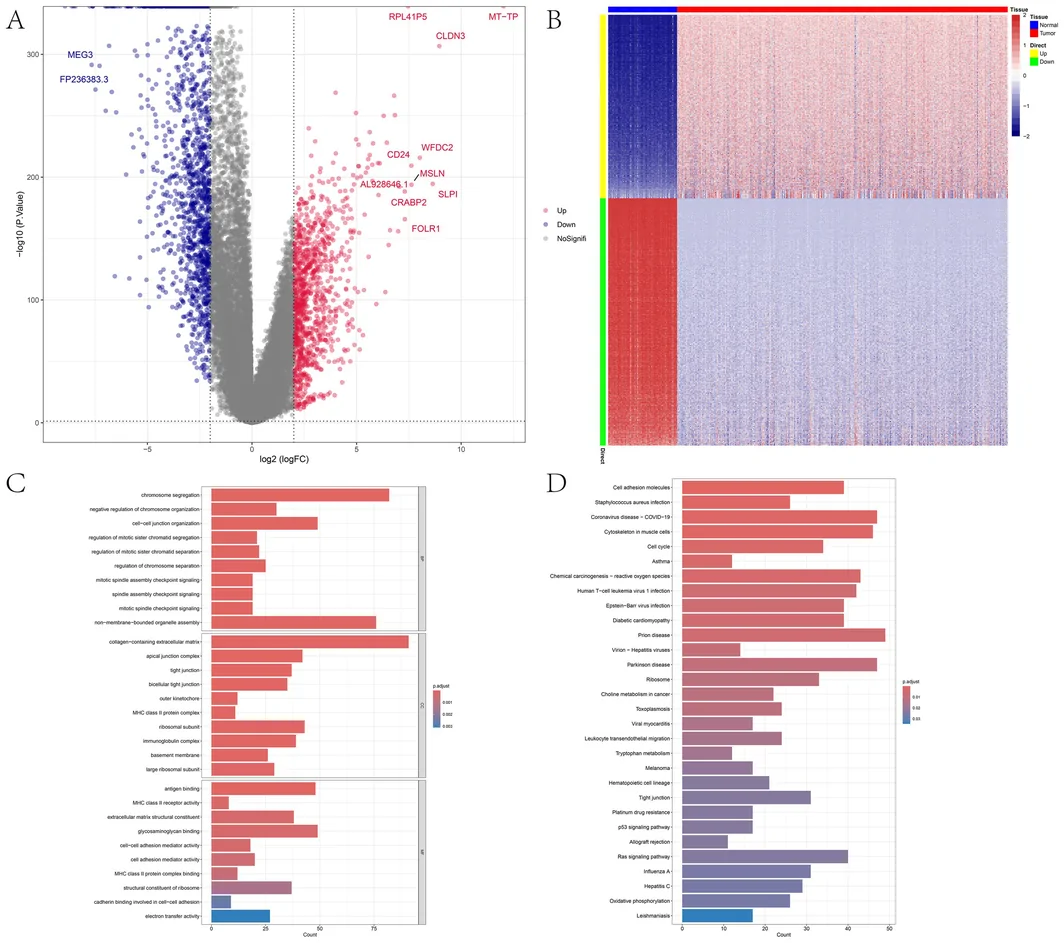
Difference and enrichment analysis. (A) Volcano map of differential genes, with blue representing downregulated differential genes and red representing up‐regulated differential genes. (B) Heat maps of differential genes, blue for low expression and red for high expression. (C, D) ClusterProfiler‐based GO‐KEGG enrichment analysis.

### 3.2 Random Survival Forest Determines Key Genes

We intersected the differentially expressed genes with the immune‐related genes from the immport database (Figure 2A), and 198 overlapping target genes were screened out in total, which were subjected to random survival forest analysis. We showed 10 genes of relative importance (Figure 2B). We further explored the prognostic value of the 10 candidate genes and confirmed that five candidates exhibited significant survival differences (*p* < 0.05) (Figure 2C–G): RELB, CXCL9, PI3, TRAC, and CXCL11. These five genes are key targets for our subsequent studies.

**FIGURE 2:**
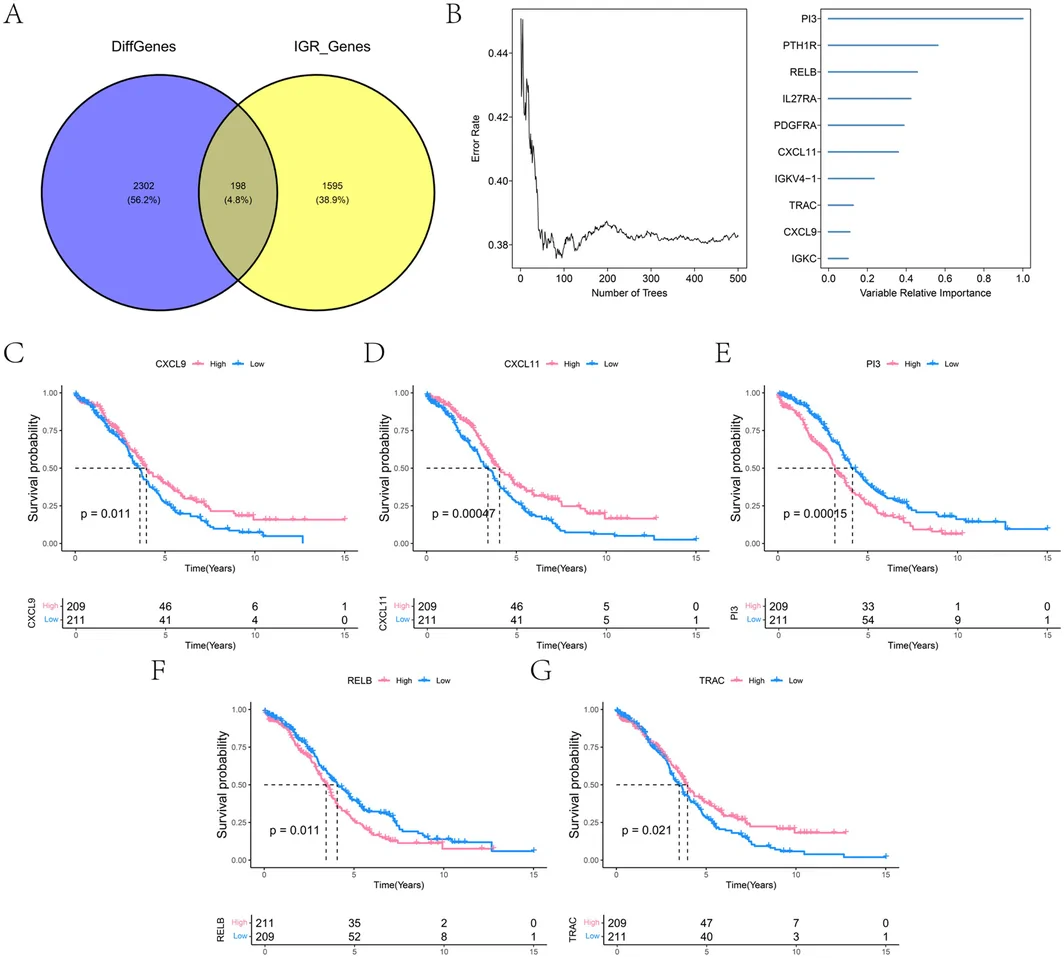
Random survival forest. (A) Wayne diagram of the intersection of differential genes and immune‐related genes. DiffGenes, differentially expressed genes; IGR, immune‐related genes. (B) The relationship between the error rate of the random forest model and the number of trees. (C–G) Survival of important genes.

### 3.3 Drug Sensitivity Analysis

Surgery plus adjuvant chemotherapy yields significant results in early‐stage OC. This research relied on drug resistance datasets retrieved from the GDSC repository. The R tool “oncoPredict” was applied to evaluate chemotherapeutic responsiveness in individual tumor specimens, as well as to explore the drug susceptibility of key genes toward conventional clinical agents. The results indicated that all five key genes were sensitive to the drug KU‐55933_1030; RELB, CXCL9, TRAC, and CXCL11 were associated with sensitivity to the chemotherapeutic agents Camptothecin_1003 and Cisplatin_1005; CXCL9, PI3, and TRAC were correlated with the sensitivity of the drug PD173074_1049. Meanwhile, CXCL9 and TRAC were related to the sensitivity of the drug Axitinib_1021. PI3 was uniquely associated with the sensitivity to the drug Vorinostat_1012 (Figure S1A–E). These findings may serve as a reference for exploring candidate therapeutic targets and provide potential clues for subsequent research on genotype‐guided precision treatment strategies, although further experimental and clinical validation are required.

### 3.4 Immune Infiltration

The TME constitutes an intricate ecosystem, which encompasses cancer‐associated fibroblasts, immunocytes, extracellular matrix components, diverse growth regulators, proinflammatory mediators, distinct physicochemical properties, and cancer cells themselves. As a key determinant of tumor progression, the TME profoundly influences clinical diagnosis, long‐term prognostic performance, and therapeutic responsiveness to routine clinical interventions (such as chemotherapy and immunotherapy). By integrating gene expression profiling with immune infiltration analysis, this study further elucidated the underlying molecular mechanisms whereby key gene expression regulates OC progression through TME‐related pathways. Compared with the low expression group of key genes, the levels of aDCs, APC_co_inhibition, APC_co_stimulation, etc. in the high expression group samples were significantly higher. Moreover, in the CXCL9, CXCL11, and TRAC high‐expression groups, marked increases were also observed in CD8+_T_cells abundance and Cytolytic activity levels (Figure S2A–E). We next explored the intrinsic association of key genes with immune cell components. The results revealed that CXCL9, TRAC, and CXCL11 presented strong positive correlations with pro‐inflammatory status; multiple distinct correlation patterns were observed among hub genes and immune cell subsets as well as immunomodulatory pathways. CXCL9 and TRAC showed a strong positive association with TIL; CXCL9 and CXCL11 presented favorable correlation with Th1_cells. Likewise, PI3 and RELB were closely linked to CCR and Macrophages, while RELB together with TRAC exerted positive relevance to T_cell_co‐stimulation. In detail, CXCL11 had marked synergistic correlation with APC_co_inhibition, and PI3 displayed prominent positive linkage to DCs. (Figure S3A–E). Correlation analyses between the identified key genes and multiple immune components were retrieved from TISIDB (Tumor Immune System Interaction Database, http://cis.hku.hk/TISIDB/index.php), covering immunomodulators, chemokines, and cell surface receptors. Data outcomes indicated that CXCL9, CXCL11, and TRAC presented close associations with diverse immune mediators; PI3 was mainly significantly correlated with chemokines; and RELB was mainly correlated with immunostimulatory factors (Figure S4A–E).

### 3.5 GSEA

Multiple critical immune signaling pathways, represented by NF‐kappa B, TNF and IL‐17, were identified as significantly enriched for the RELB gene via GSEA evaluation (Figure S5A). CXCL9 exhibited significant enrichment within diverse biological signaling cascades, covering helper T cell subtype maturation, Toll‐like receptor‐mediated signal transduction, and additional immune regulatory routes (Figure S5B). PI3 was enriched in Cytokine‐cytokine receptor interaction, Epstein–Barr virus infection, Phagosome, and other signaling pathways (Figure S5C). TRAC was enriched in Chemokine signaling pathway, Osteoclast differentiation, NOD‐like receptor regulatory cascade and several additional biological signaling axes (Figure S5D). CXCL11 was overrepresented in Cell adhesion molecules, Cytosolic DNA‐sensing cascade, RIG‐I‐like receptor signaling routes, and other signaling pathways (Figure S5E). GSEA uncovered strong enrichment patterns of key genes in immune and inflammation‐associated biological pathways, suggesting that these molecules jointly modulate disease advancement and clinical outcomes of OC via coordinately modulating distinct features of the TIME. In particular, the enrichment of PI3 and CXCL11 in the antiviral innate immunity pathway implies a potential unique role of virus‐related immune response mechanisms in OC, thereby providing a new avenue for research into the immune evasion mechanisms of this malignancy.

### 3.6 GSVA

GSVA demonstrated significant heterogeneity in pathway enrichment patterns among the key genes. RELB and PI3 were co‐enriched in the TNFA_SIGNALING_VIA_NFKB pathway, suggesting their potential role in chemotherapy resistance (Figure 3A,C). CXCL9 and TRAC converged on the allograft_rejection pathway, identifying CXCL9 and TRAC as potential biomarkers for predicting immunotherapy response (Figure 3B,D). Strong enrichment of CXCL11 was detected in signature sets associated with type I and type II interferon response pathways, indicating its ability to activate DC‐dependent antigen processing and the cytotoxic activity of NK cells, collectively enhancing immune‐mediated tumor clearance (Figure 3E). PI3 uniquely linked COAGULATION with inflammatory signaling and was enriched in glycolysis and cholesterol homeostasis pathways, implying its dual role in promoting thrombosis and abnormal angiogenesis, which may serve as a predictor of resistance to anti‐angiogenic drugs (Figure 3C). Concurrently, we identified that key genes RELB, CXCL9, and TRAC co‐activate the INFLAMMATORY_RESPONSE pathway, indicating the presence of an NF‐kappa B/cytokine‐driven immunosuppressive microenvironment in OC (Figure 3A,B,D).

**FIGURE 3:**
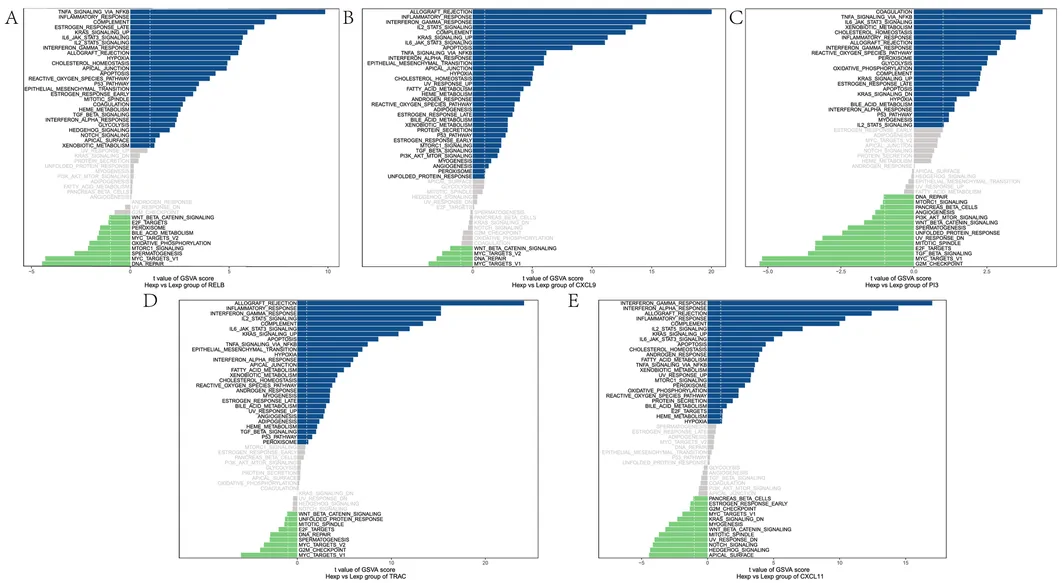
GSVA analysis of key genes. (A–E) GSVA analysis of key genes. The signal pathway involved in high expression of blue expressed genes and the signal pathway involved in low expression genes were represented by green. The background gene set was hallmark.

### 3.7 Transcriptional Regulatory Network, Noncoding RNA Network Related to Key Genes

These key genes were designated as the research‐focused gene collection in our analysis and confirmed to be modulated by common regulatory mechanisms, including diverse transcription factors. Thus, we evaluated the enrichment levels of these transcriptional regulators using cumulative recovery curves. Combined analysis of Motif‐TF annotation and key gene screening demonstrated that cisbp__M5082 ranked first in terms of normalized enrichment values (Figure 4A,B), with an NES of 8.36. By employing the mircode database, we executed reverse prediction of key genes, finding 64 miRNAs and 100 mRNA‐miRNA pairs, visualized through cytoscape (Figure 4C).

**FIGURE 4:**
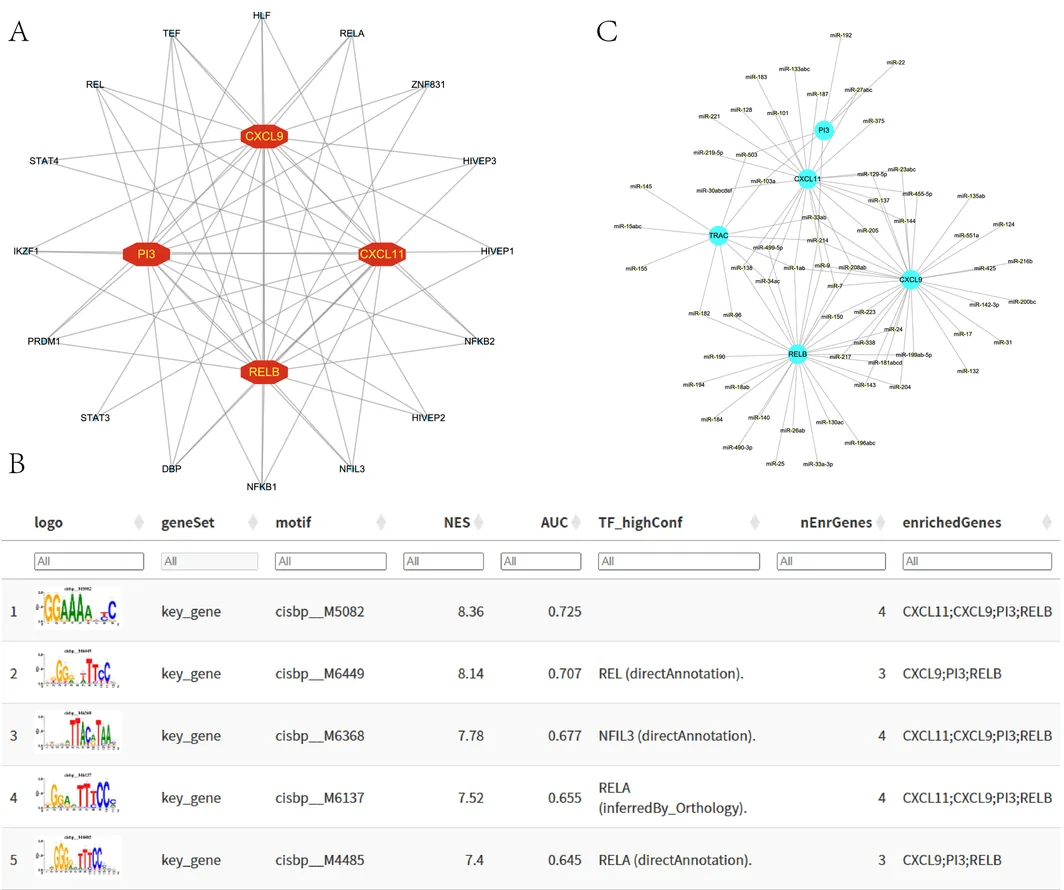
Key gene‐related transcriptional regulation and miRNA networks. (A) Transcriptional regulatory networks of key genes, with red representing key genes and blue representing transcription factors. (B) All the enriched motifs and corresponding transcription factors of key genes were displayed. (C) miRNA networks of key genes, with blue representing mRNA and yellow representing miRNA.

### 3.8 Single‐Cell Data Processing and Annotation

Given the data reliability across diverse sample sets, cellular populations harboring fewer than 500 detected genetic features were excluded from subsequent analysis. Subsequently, the Doublet Finder algorithm was applied to eliminate cell doublets, ultimately preserving 46,235 valid cellular units in total. Following sequential normalization, homogenization, PCA dimensionality reduction and Harmony batch correction, the uniform manifold approximation and projection (UMAP) was leveraged to perform dimension compression, and each subpopulation is annotated. We classified cellular clusters into seven distinct cell lineages: T cells, Fibroblasts, Monocytes, Epithelial cells, Endothelial cells, B cells, and SMC (Figure S6A,B). The bubble plot displaying canonical biomarkers of the seven cell types (Figure S5C) and the cell ratio bar chart corresponding to the disease control group (Figure S6D). Next, the DotPlot and Feature Plot functions in the Seurat R package were utilized to map the expression patterns of core genes at single‐cell resolution (Figure S7A,B). The results showed that the key genes CXCL9 and CXCL11 had higher expression levels in monocytes, TRAC had higher expression levels in T cells, and PI3 had higher expression levels in epithelial cells.

### 3.9 Immune Metabolism Pathway

We applied AUCell to estimate the transcriptional activity scores of genes involved in immune‐metabolism‐related pathways at the single‐cell level and visualized the expression differences of key genes in these pathways using a bubble plot. The results showed that TRAC, CXCL9, and CXCL11 exhibited high activity in the allograft rejection signaling pathway, which has been shown to involve immunometabolic reprogramming of proinflammatory macrophages with elevated glycolytic capacity (Chen et al. 2024). These genes also showed high activity in both type I (interferon‐alpha) and type II (interferon‐gamma) interferon response pathways. Type I interferons directly reprogram tumor cell metabolism by activating oxidative phosphorylation for ATP production in an ISG15‐dependent manner, while type II interferon (IFN‐γ) is closely linked to glycolysis‐dependent metabolic reprogramming and functional plasticity in immune cells (Wang et al. 2018; Zhou et al. 2024). PI3 showed high activity in the glycolysis and mtorc1_signaling immune pathways, both of which are central regulators of immunometabolism. Furthermore, CXCL9 and CXCL11 exhibited high activity in the tnfa_signaling_via_nfkb immune pathway, which intersects with metabolic regulation and connects inflammatory signaling to metabolic reprogramming (Capece et al. 2022) (Figure 5A).

**FIGURE 5:**
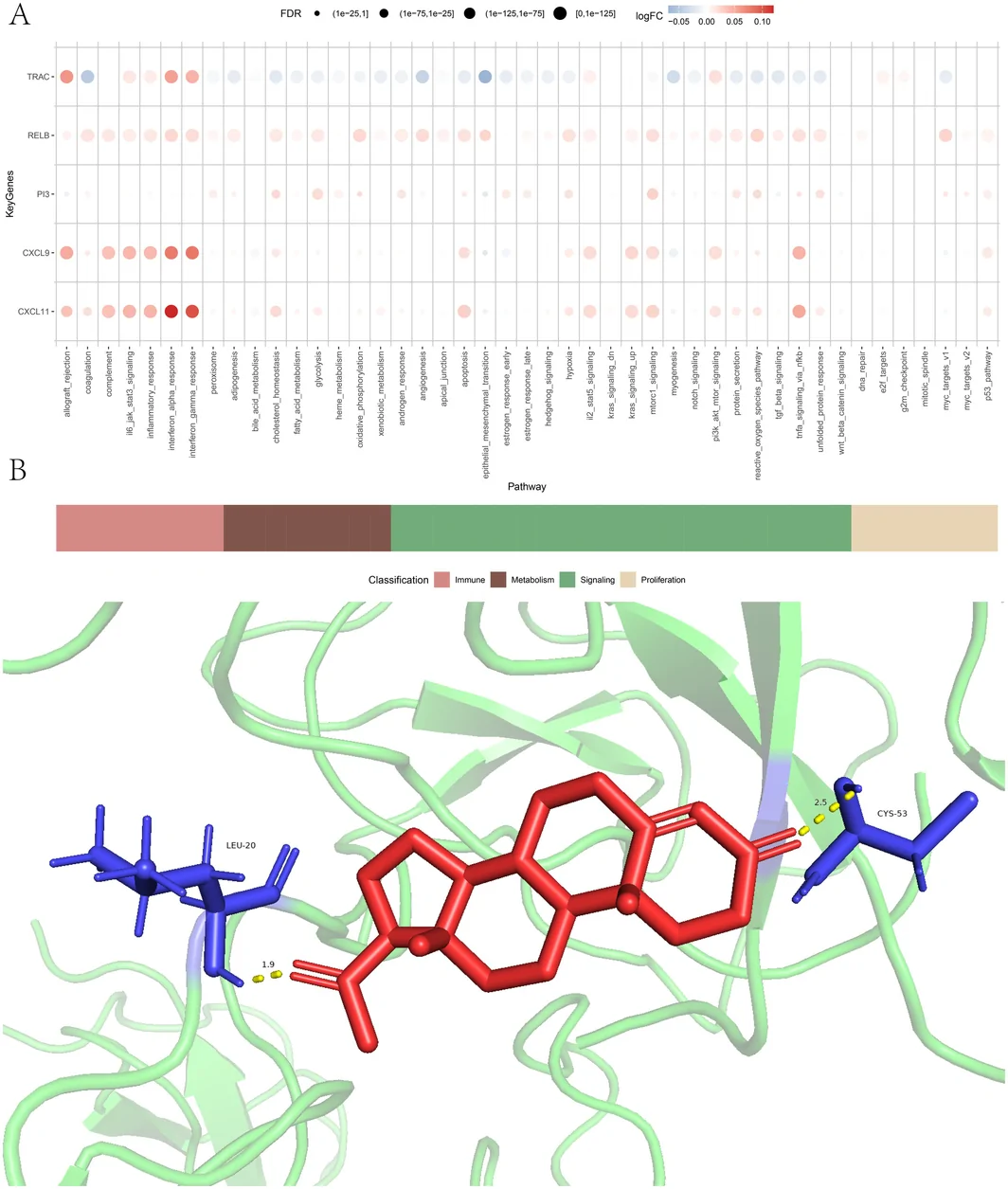
Differences of key genes and immune metabolic pathways and molecular docking. (A) Differences in the activity of key genes and immune metabolism, with low expression in blue and high expression in red. (B) Molecular docking networks of key genes.

### 3.10 Molecular Docking Results

Molecular docking simulations indicated that the binding affinity energy between OC‐related PI3 protein and 6atu‐PROGESTERONE reached −6.35 kcal/mol, and there is a strong affinity between the two. PI3 and 6atu‐PROGESTERONE form interactions mediated by Leu‐20 and Cys‐53 amino acid residues (Figure 5B). This suggests that the combination of the two may have clinical significance as a candidate compound against OC.

### 3.11 Validation of Core Gene Expression in Ovarian Cancer Cell Lines

To further validate the five core genes at the cellular level, we first conducted qRT‐PCR assays to determine the relative mRNA expression of PI3, RELB, and TRAC in normal ovarian epithelial cells (HOSEpiC) and two ovarian cancer cell lines (SKOV3 and A2780). Since the other two core genes, CXCL9 and CXCL11, are common chemokines, no further determination of gene expression was carried out. As shown in Figure 6A, compared to HOSEpiC cells, the expression of RELB in SKOV3 and A2780 cells was significantly increased (*p* < 0.01), suggesting that RELB may play a role in carcinogenesis in ovarian cancer. In contrast, the expression of PI3 in A2780 cells was slightly higher than that in HOSEpiC cells (*p* < 0.05), while there was no significant difference in SKOV3 cells compared to the normal control group, indicating that its expression is cell line‐specific (Figure 6B). It is noteworthy that the expression of TRAC between normal cell lines and cancer cell lines showed no significant difference (Figure 6C), which is consistent with its main expression in immune cells rather than tumor epithelial cells. Therefore, in the subsequent analysis, we chose RELB for further functional verification.

**FIGURE 6:**
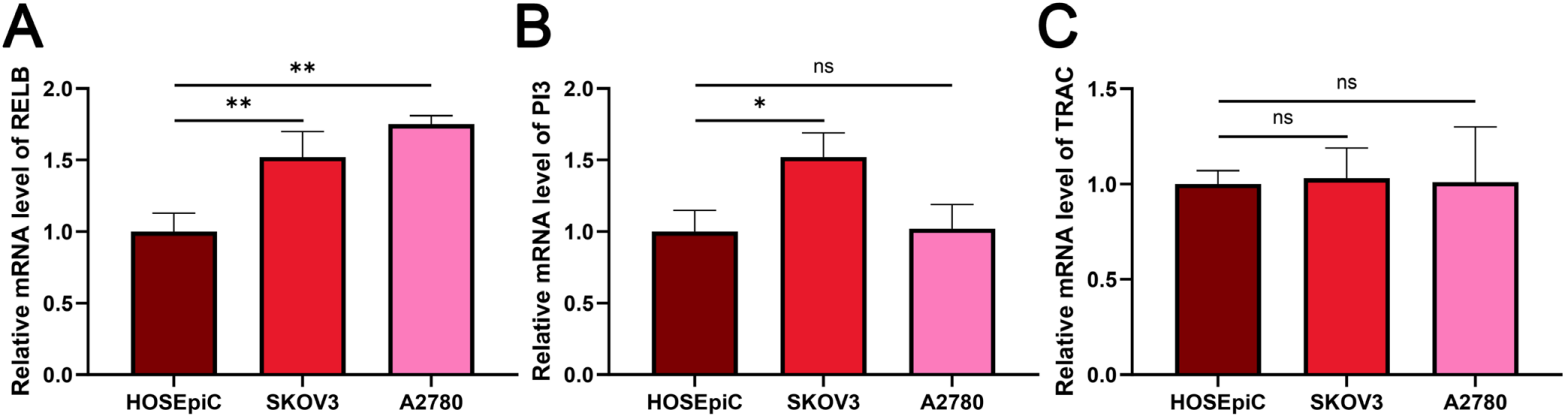
Validation of core gene expression in ovarian cancer cell lines. (A–C) qRT‐PCR was used to determine the expression of RELB (A), PI3 (B), and TRAC (C) in ovarian cancer cell lines. ns represents No significant difference, *p < 0.05, **p < 0.01.

### 3.12 RELB Promotes Malignant Phenotypes in Ovarian Cancer Cells

To validate the functional role of RELB in ovarian cancer cells, we performed siRNA‐mediated knockdown in A2780 cells. qRT‐PCR analysis confirmed that RELB mRNA expression was significantly reduced in the si‐RELB group compared with the Control and si‐NC groups, indicating successful knockdown (Figure 7A, *p* < 0.05). We then evaluated the effect of RELB knockdown on cell proliferation using the CCK‐8 assay. As shown in Figure 7B, RELB knockdown significantly inhibited the proliferative capacity of A2780 cells over time (*p* < 0.05). Furthermore, flow cytometry analysis revealed that RELB silencing markedly increased the apoptotic rate compared with the Control and si‐NC groups (Figure 7C, *p* < 0.05). Collectively, these results demonstrate that RELB functions as a pro‐survival and pro‐proliferative factor in A2780 cells, and its downregulation impairs OC cell growth while promoting apoptosis. Further analysis was conducted on the effects of RELB on cell migration and invasion. The results showed that after knockdown of RELB, both cell migration and invasion abilities significantly decreased (Figure 7D,E, *p* < 0.05).

**FIGURE 7:**
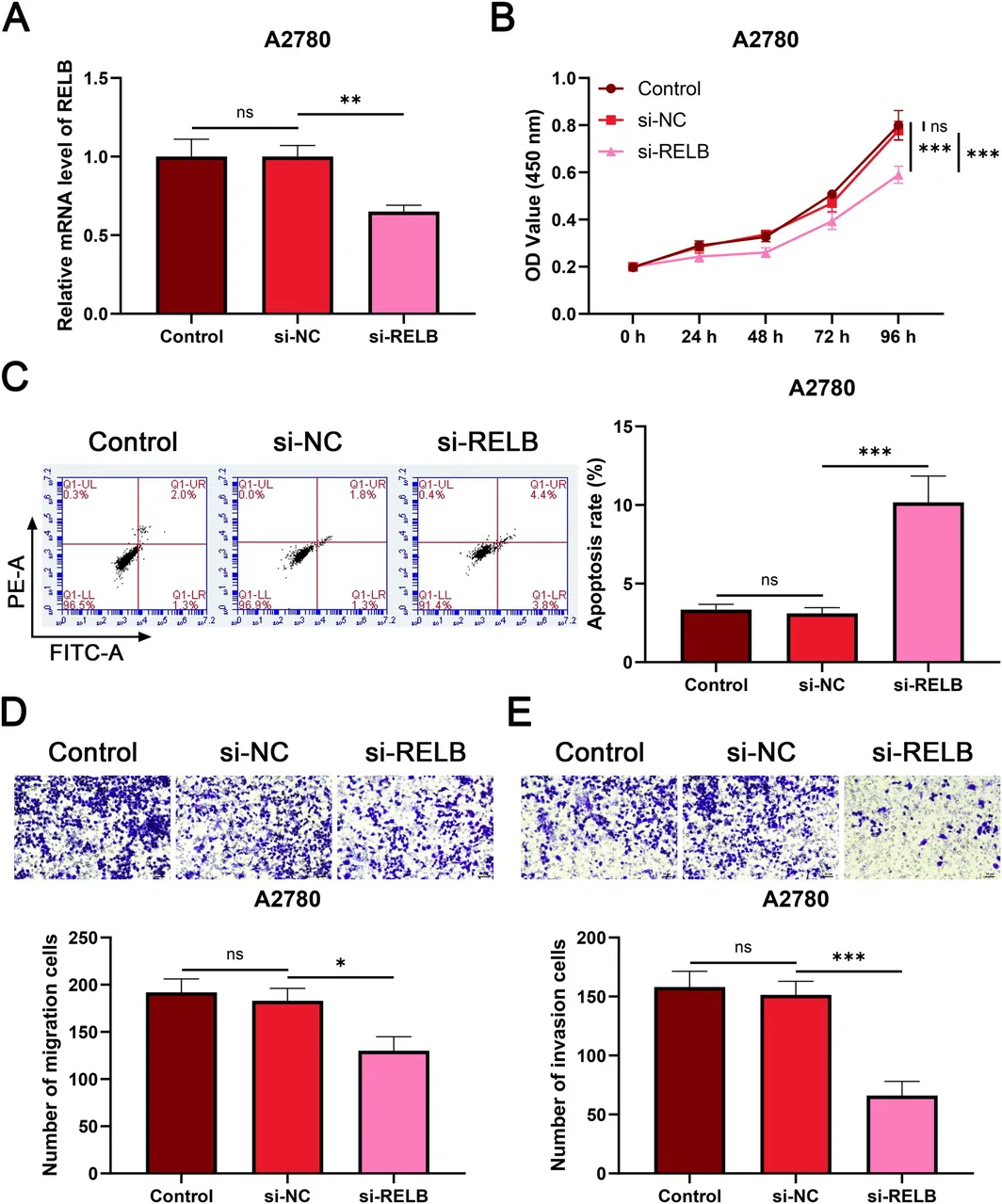
RELB promotes malignant phenotypes in ovarian cancer cells. (A) Determine the knockdown efficiency of RELB by qRT‐PCR. (B) Assess the effect of RELB knockdown on cell proliferation using CCK‐8. (C) Analyze cell apoptosis by flow cytometry. (D) Investigate the impact of RELB knockdown on cell migration through Transwell assay. (E) Examine the influence of RELB knockdown on cell invasion via Transwell assay. ns represents No significant difference, *p < 0.05, **p < 0.01, ***p < 0.001.

## 4 Discussion

OC is still one of the primary drivers of morbidity and death among women. It represents a highly malignant condition frequently identified at late clinical stages but is highly sensitive to platinum‐based chemotherapy in its early stages. However, recurrent disease commonly occurs in most patients who receive initial surgery plus chemotherapy, highlighting the urgent need to develop new treatment strategies (Siegel et al. 2021). Recent years have witnessed major breakthroughs in targeted therapy for OC, including PARPis and anti‐angiogenic agents. However, significant challenges persist, such as drug resistance, high relapse rates, and the limited efficacy of CPI (Xu et al. 2026). Thus, unraveling the molecular mechanisms of OC—particularly the regulatory network of the TIME—is of great importance for discovering novel biomarkers and therapeutic targets.

Through combining single‐cell profiling with bulk RNA sequencing datasets, the present research successfully screened five hub genes (RELB, CXCL9, CXCL11, PI3, and TRAC) that are closely associated with immune‐ and inflammation‐related pathways. We further revealed their potential roles in OC progression, immune infiltration, metabolic reprogramming, and treatment response.

In this study, a random survival forest analysis was fitted to the overlapping region between dysregulated genetic transcripts and immunity‐associated genes, leading to the identification of five key genes (RELB, CXCL9, CXCL11, PI3, and TRAC) closely associated with clinical outcomes in OC sufferers. Survival analysis demonstrated that the transcript abundance in the five genes was strongly linked to patient prognosis (*p* < 0.05). CXCL9 and CXCL11, pivotal CXC chemokine subfamily molecules, serve as key mediators in establishing a T cell inflamed antitumor phenotype (Reschke et al. 2024).

Our immune infiltration analysis further strengthened this conclusion. Specifically, the high‐expression groups of CXCL9, CXCL11, and TRAC exhibited markedly elevated CD8+_T_cells levels and Cytolytic activity. Furthermore, CXCL9 and TRAC exhibited strong positive correlations toward TIL, while CXCL9 and CXCL11 presented favorable relevance toward Th1_cells. This finding is in agreement with previous evidence that STAT1‐driven chemokine molecules CXCL9, CXCL10, and CXCL11 are closely correlated with the magnitude of intratumoral CD8^+^ T cell infiltration (Du et al. 2025).

CXCL9 exerts a critical function in anti‐cancer immune responses via regulating immune cell recruitment and functional activation, and has potential synergistic effects with immune checkpoint blockade therapy (Wang, Zhao, et al. 2025). Notably, TRAC, the T‐cell receptor alpha chain constant region gene, showed a strong correlation with T cells in this study. This aligns with the documented prognostic value pertaining to T‐cell receptor profiling traits among OC cases, as previous studies have shown that TCR profiling signatures of intratumoral immune lymphocytes influence patient outcomes (Bentham et al. 2021).

RELB, a core component governing atypical NF‐κB signal cascades, showed a significantly positive correlation with CCR and Macrophages in this study, as well as a positive correlation with T_cell_co‐stimulation. Previous studies have demonstrated that the TWEAK‐Fn14‐RELB signaling pathway is enriched in OC following chemotherapy treatment, promotes cancer stem‐cell‐like features, and contributes to post‐chemotherapy relapse (Holmberg et al. 2023). Evidence reveals that the NF‐kappa B transcription factors RELA and RELB are activated by stimuli originating from the ovarian tumor microenvironment, whereupon they cooperatively foster proliferative expansion, chemotherapeutic resistance, and sustained viability (House et al. 2017). The co‐enrichment of RELB with the TNFA_SIGNALING_VIA_NFKB pathway observed in this study is highly consistent with these mechanistic findings, suggesting that RELB may participate in the regulation of chemotherapy resistance in OC through the non‐canonical NF‐kappa B signaling pathway.

GSEA and GSVA analyses further demonstrated the functional heterogeneity of the identified key genes across various signaling pathways. RELB, CXCL9, and TRAC co‐activated inflammatory response pathways, whereas RELB and PI3 exhibited co‐enrichment in the TNFA_SIGNALING_VIA_NFKB pathway; CXCL11 was specifically enriched in the interferon response pathway, indicating that it may facilitate immune‐mediated tumor elimination by promoting DC‐dependent antigen processing and NK cell cytotoxic activity. Consistent with previous studies, CXCL11, as a STAT1 target gene, has been shown to correlate significantly with CD8A transcript levels in HGSOC (Au et al. 2016). The high enrichment of PI3 in glycolysis and mtorc1_signaling immune pathways has significant functional implications. Consistent with the current understanding, altered metabolic patterns serve as a vital oncogenic feature of OC, enabling tumor progression, immune evasion, and drug resistance (Kumari et al. 2025). Reprogramming of glucose metabolism promotes chemoresistance in tumors via overexpression of metabolic enzymes in aerobic glycolysis and the pentose phosphate pathway. As a result, key molecules in these pathways are now being recognized as novel drug targets (Liu et al. 2025). PI3 is uniquely positioned at the intersection of COAGULATION and inflammatory signaling, and is also enriched in the cholesterol homeostasis pathway. These features suggest a dual role for PI3 in promoting thrombosis and abnormal angiogenesis, which could be exploited as a predictor of resistance to anti‐angiogenic drugs.

In addition, AUCell was utilized to assess the functional activity pertaining to immune metabolic pathways in single cells. TRAC, CXCL9, and CXCL11 displayed high activity in allograft_rejection, interferon response pathways, whereas PI3 showed high activity in glycolysis and mtorc1_signaling immune pathways. Single‐cell resolution further revealed cell‐type‐specific expression patterns: CXCL9 and CXCL11 were enriched in monocytes, TRAC in T cells, and PI3 in epithelial cells. These results implicate that the five key genes may jointly influence OC progression and prognosis through synergistic regulation of distinct facets of the TIME. Of note, the enrichment of PI3 and CXCL11 in antiviral innate immune pathways points to a distinctive role for virus‐associated immune responses in OC, offering a novel perspective for understanding immune evasion. Moreover, CXCL9 and CXCL11 exhibited robust activity in the TNFA_SIGNALING_VIA_NFKB immune pathway. Given the previously established bidirectional regulatory relationship between chemokines and NF‐kappa B signaling, these molecules may co‐modulate inflammatory processes through a positive feedback loop.

Based on GDSC drug sensitivity data, the five key genes showed distinct sensitivity profiles to multiple chemotherapeutic and targeted agents. All five were sensitive to the ATM inhibitor KU‐55933. RELB, CXCL9, TRAC, and CXCL11 were associated with sensitivity to Cisplatin and Camptothecin; CXCL9 and TRAC correlated with Axitinib sensitivity; and PI3 uniquely correlated with Vorinostat sensitivity. Collectively, these findings suggest that the expression patterns of the above key genes could act as promising molecular markers to guide personalized treatment for OC. Notably, the unique PI3‐Vorinostat correlation, coupled with PI3 enrichment in glucose metabolism and COAGULATION pathways, suggests PI3 as a potential target for combining epigenetic therapy with metabolic modulation.

Molecular docking further showed that PI3 bound tightly to 6atu‐PROGESTERONE with a binding energy of −6.35 kcal/mol, interacting through Leu‐20 and Cys‐53 residues. Progesterone exerts context‐dependent effects in OC: It can shield OC cells against Cisplatin‐triggered suppression through progesterone receptor membrane subunits 1/2 and AKT cascade signaling (Zhu et al. 2013), while progesterone receptor signaling promotes quiescence and OC cell phenotypes through the DREAM complex within p53‐altered fallopian tube systems (Mauro et al. 2021). The high‐affinity binding of PI3 to 6atu‐PROGESTERONE implies that this compound may regulate metabolic and inflammatory pathways in OC cells by targeting PI3, yet its definitive biological activity awaits further functional validation.

Cell function experiments have demonstrated that the knockdown of RELB can significantly affect the malignant phenotype of OC cells. This is consistent with previous studies. For instance, Olazaba et al. also proved that the knockdown of RELB could inhibit the recurrence of HGSOC and drive integrin‐mediated stress tolerance (Lujano‐Olazaba et al. 2026). The same was true in the study conducted by Holmberg et al. (2023) However, there are relatively few studies on RELB in the ocular cavity, and the mechanism of its action in the ocular cavity still needs to be further explored.

This study has several limitations. To begin with, all analyses are based on retrospective data from public databases (TCGA, GEO, and GTEx) without prospective external validation. Batch effects and selection bias may exist. Second, the findings are primarily computational and lack in vitro/in vivo functional validation. Although molecular docking predicts high affinity between PI3 and 6atu‐PROGESTERONE, experimental confirmation via knockout/overexpression and animal models is needed. Third, the single‐cell data derive from limited samples (5 controls and 7 tumors), potentially underrepresenting the full cellular diversity of OC. Fourth, immune infiltration inferences from transcriptomic data require direct validation by multiplex immunofluorescence or flow cytometry. Finally, although chemokine‐ NF‐kappa B bidirectional regulation is documented, the specific molecular details of this positive feedback loop in OC require further investigation. Fifth, the computational prediction values of the five prognostic genes for the sensitivity of KU‐55933 require further experimental research. In the future, by using chemical inhibition or CRISPR‐mediated genetic perturbation combined with transcriptome analysis, it will be significant to clarify the specific regulatory circuits of this compound in regulating the target gene network in ovarian cancer cell models. Finally, the mechanism of these five core genes in OC still needs to be further explored in cell and animal experiments.

## 5 Conclusions

In summary, integrative multi‐omic and single‐cell analyses identified five key genes—RELB, CXCL9, CXCL11, PI3, and TRAC—that were closely associated with the immune microenvironment of OC. Functional enrichment and immune infiltration analyses further supported their potential involvement in OC progression. Among these genes, RELB was highly expressed in OC cells, and its knockdown significantly inhibited proliferation, migration, and invasion while promoting apoptosis, supporting its potential pro‐tumorigenic role. Overall, these findings identify candidate biomarkers and therapeutic targets for OC, although further validation in independent patient cohorts and mechanistic studies is required.

## Author Contributions

W.L.: Conceptualization, data curation, formal analysis, investigation, funding acquisition, and writing – original draft. X.F.: Supervision, validation, visualization, project administration, and software. Y.F.: Methodology, project administration, resources, software, and writing – review and editing.

## Funding

The work was supported by the Research Project of Jiangsu Cancer Hospital (No. XHZD202503).

## Ethics Statement

The authors have nothing to report.

## Conflicts of Interest

The authors declare no conflicts of interest.

## Supporting information


**Figure S1:** Correlation of drug sensitivity. (A–E) Sensitivity analysis of key genes and chemotherapy drugs.
**Figure S2:** Analysis of immune differences. (A–E) Differences in immune cell content of key genes between high and low samples.
**Figure S3:** Immune correlation analysis. (A–E) Correlation between key gene immunity and epidemic cells.
**Figure S4:** Relationship between key genes and immune factors. (A–E) Correlation of key genes with chemokine, Immunoinhibitor, Immunostimulator, MHC, and receptor.
**Figure S5:** GSEA analysis of key genes. (A–E) Key genes involved in the KEGG signaling pathway, as well as pathway regulation and involved genes.
**Figure S6:** Annotation of cells. (A) We divided the cells into 12 clusters by UMAP algorithm based on the significant components available in PCA. (B) Cell annotation of 12 clusters, 12 clusters were annotated into 7 cell types, namely, T cells, Fibroblasts, Monocytes, Epithelial cells, Endothelial cells, B cells, and SMC. (C) Bubble plot of 7 types of cells and cell markers. (D) The difference in the proportion of 7 types of cells in the two samples.
**Figure S7:** Expression of single cells. (A) Scatterplot of key gene expression profiles in single cells. (B) Bubble map of key gene expression profiles in single cells. Blue indicates low expression and red indicates high expression.

## Data Availability

The data that support the findings of this study are available on request from the corresponding author. The data are not publicly available due to privacy or ethical restrictions.

## References

[cbdd70409-bib-0001] Au, K. K. , C. Le Page , R. Ren , et al. 2016. “STAT1‐Associated Intratumoural T(H)1 Immunity Predicts Chemotherapy Resistance in High‐Grade Serous Ovarian Cancer.” Journal of Pathology and Clinical Research 2, no. 4: 259–270. 10.1002/cjp2.55. PMC5129574 27917296

[cbdd70409-bib-0002] Bentham, R. , K. Litchfield , T. B. K. Watkins , et al. 2021. “Using DNA Sequencing Data to Quantify T Cell Fraction and Therapy Response.” Nature 597, no. 7877: 555–560. 10.1038/s41586-021-03894-5. 34497419

[cbdd70409-bib-0003] Bogani, G. , K. N. Moore , I. Ray‐Coquard , et al. 2025. “Incorporating Immune Checkpoint Inhibitors in Epithelial Ovarian Cancer.” Gynecologic Oncology 193: 30–40. 10.1016/j.ygyno.2024.12.011. 39764856

[cbdd70409-bib-0004] Bryant, A. , S. Hiu , P. T. Kunonga , et al. 2022. “Impact of Residual Disease as a Prognostic Factor for Survival in Women With Advanced Epithelial Ovarian Cancer After Primary Surgery.” Cochrane Database of Systematic Reviews 9, no. 9: Cd015048. 10.1002/14651858.CD015048.pub2. PMC9512080 36161421

[cbdd70409-bib-0005] Capece, D. , D. Verzella , I. Flati , P. Arboretto , J. Cornice , and G. Franzoso . 2022. “NF‐κB: Blending Metabolism, Immunity, and Inflammation.” Trends in Immunology 43, no. 9: 757–775. 10.1016/j.it.2022.07.004. 35965153

[cbdd70409-bib-0006] Chang, X. , Y. Zheng , and K. Xu . 2024. “Single‐Cell RNA Sequencing: Technological Progress and Biomedical Application in Cancer Research.” Molecular Biotechnology 66, no. 7: 1497–1519. 10.1007/s12033-023-00777-0. PMC11217094 37322261

[cbdd70409-bib-0007] Chen, Z. , Y. Li , Y. Niu , et al. 2024. “MEK1/2‐PKM2 Pathway Modulates the Immunometabolic Reprogramming of Proinflammatory Allograft‐Infiltrating Macrophages During Heart Transplant Rejection.” Transplantation 108, no. 5: 1127–1141. 10.1097/tp.0000000000004899. PMC11042528 38238904

[cbdd70409-bib-0008] Colombo, N. , and J. A. Ledermann . 2021. “Updated Treatment Recommendations for Newly Diagnosed Epithelial Ovarian Carcinoma From the ESMO Clinical Practice Guidelines.” Annals of Oncology 32, no. 10: 1300–1303. 10.1016/j.annonc.2021.07.004. 34293462

[cbdd70409-bib-0009] Di, Z. , M. Li , and P. Li . 2025. “METTL3 Promotes Ovarian Cancer Progression Through YTHDF2‐Dependent Degradation of GATA4.” Pathology, Research and Practice 275: 156224. 10.1016/j.prp.2025.156224. 40945198

[cbdd70409-bib-0010] Du, Y. , B. Zheng , X. Wang , et al. 2025. “Influencing Factors of Health Utility Values in Older Adult People With Normal Cognition or Mild Cognitive Impairment: A Cross‐Sectional Survey.” Frontiers in Public Health 13: 1538665. 10.3389/fpubh.2025.1538665. PMC11936783 40144983

[cbdd70409-bib-0011] Gaillard, S. , C. Lacchetti , D. K. Armstrong , et al. 2025. “Neoadjuvant Chemotherapy for Newly Diagnosed, Advanced Ovarian Cancer: ASCO Guideline Update.” Journal of Clinical Oncology 43: JCO2402589. 10.1200/JCO-24-02589. PMC11934100 39841949

[cbdd70409-bib-0012] Holmberg, R. , M. Robinson , S. F. Gilbert , et al. 2023. “TWEAK‐Fn14‐RelB Signaling Cascade Promotes Stem Cell‐Like Features That Contribute to Post‐Chemotherapy Ovarian Cancer Relapse.” Molecular Cancer Research 21, no. 2: 170–186. 10.1158/1541-7786.Mcr-22-0486. PMC9890141 36214671

[cbdd70409-bib-0013] House, C. D. , E. Jordan , L. Hernandez , et al. 2017. “NFκB Promotes Ovarian Tumorigenesis via Classical Pathways That Support Proliferative Cancer Cells and Alternative Pathways That Support ALDH(+) Cancer Stem‐Like Cells.” Cancer Research 77, no. 24: 6927–6940. 10.1158/0008-5472.Can-17-0366. PMC5732863 29074539

[cbdd70409-bib-0014] Jitmana, K. , J. I. Griffiths , S. Fereday , A. DeFazio , D. Bowtell , and F. R. Adler . 2024. “Mathematical Modeling of the Evolution of Resistance and Aggressiveness of High‐Grade Serous Ovarian Cancer From Patient CA‐125 Time Series.” PLoS Computational Biology 20, no. 5: e1012073. 10.1371/journal.pcbi.1012073. PMC11164342 38809938

[cbdd70409-bib-0015] Kumari, S. , S. Gupta , A. Jamil , D. Tabatabaei , and S. Karakashev . 2025. “Exploring Metabolic Approaches for Epithelial Ovarian Cancer Therapy.” Journal of Cellular Physiology 240, no. 1: e31495. 10.1002/jcp.31495. PMC12918922 39676338

[cbdd70409-bib-0016] Li, W. , K. Zhang , W. Wang , et al. 2024. “Combined Inhibition of HER2 and VEGFR Synergistically Improves Therapeutic Efficacy via PI3K‐AKT Pathway in Advanced Ovarian Cancer.” Journal of Experimental & Clinical Cancer Research 43, no. 1: 56. 10.1186/s13046-024-02981-5. PMC10895844 38403634

[cbdd70409-bib-0017] Liu, C. , D. Liu , F. Wang , et al. 2022. “Identification of a Glycolysis‐ and Lactate‐Related Gene Signature for Predicting Prognosis, Immune Microenvironment, and Drug Candidates in Colon Adenocarcinoma.” Frontiers in Cell and Developmental Biology 10: 971992. 10.3389/fcell.2022.971992. PMC9445192 36081904

[cbdd70409-bib-0018] Liu, C. , W. Liu , J. Huang , et al. 2025. “Metabolic Reprogramming Shapes the Progression and Therapeutic Landscape of Ovarian Cancer.” Cancer Management and Research 17: 1707–1722. 10.2147/cmar.S538281. PMC12374702 40861078

[cbdd70409-bib-0019] Lujano‐Olazaba, O. , M. Robinson , G. J. Jordan , et al. 2026. “RelB Drives Integrin‐Mediated Stress Tolerance and Relapse in High‐Grade Serous Ovarian Cancer.” Cell Reports 45, no. 7: 117650. 10.1016/j.celrep.2026.117650. PMC13464571 42400907

[cbdd70409-bib-0020] Mauro, L. J. , M. I. Seibel , C. H. Diep , et al. 2021. “Progesterone Receptors Promote Quiescence and Ovarian Cancer Cell Phenotypes via DREAM in p53‐Mutant Fallopian Tube Models.” Journal of Clinical Endocrinology and Metabolism 106, no. 7: 1929–1955. 10.1210/clinem/dgab195. PMC8499172 33755733

[cbdd70409-bib-0021] Meng, L. , and S. Sun . 2024. “Single‐Cell RNA Sequencing Reveals the Change in Cytotoxic NK/T Cells, Epithelial Cells and Myeloid Cells of the Tumor Microenvironment of High‐Grade Serous Ovarian Carcinoma.” Discover Oncology 15, no. 1: 417. 10.1007/s12672-024-01290-9. PMC11383903 39249551

[cbdd70409-bib-0022] Moore, K. N. , A. Angelergues , G. E. Konecny , et al. 2023. “Mirvetuximab Soravtansine in FRα‐Positive, Platinum‐Resistant Ovarian Cancer.” New England Journal of Medicine 389, no. 23: 2162–2174. 10.1056/NEJMoa2309169. 38055253

[cbdd70409-bib-0023] Reschke, R. , A. H. Enk , and J. C. Hassel . 2024. “Chemokines and Cytokines in Immunotherapy of Melanoma and Other Tumors: From Biomarkers to Therapeutic Targets.” International Journal of Molecular Sciences 25, no. 12: 6532. 10.3390/ijms25126532. PMC11203481 38928238

[cbdd70409-bib-0024] Siegel, R. L. , K. D. Miller , H. E. Fuchs , and A. Jemal . 2021. “Cancer Statistics, 2021.” CA: A Cancer Journal for Clinicians 71, no. 1: 7–33. 10.3322/caac.21654. 33433946

[cbdd70409-bib-0025] Sun, F. , H. Li , D. Sun , et al. 2025. “Single‐Cell Omics: Experimental Workflow, Data Analyses and Applications.” Science China. Life Sciences 68, no. 1: 5–102. 10.1007/s11427-023-2561-0. 39060615

[cbdd70409-bib-0026] Tamura, N. , N. Shaikh , D. Muliaditan , et al. 2020. “Specific Mechanisms of Chromosomal Instability Indicate Therapeutic Sensitivities in High‐Grade Serous Ovarian Carcinoma.” Cancer Research 80, no. 22: 4946–4959. 10.1158/0008-5472.Can-19-0852. 32998996

[cbdd70409-bib-0027] Therachiyil, L. , A. Anand , A. Azmi , A. Bhat , H. M. Korashy , and S. Uddin . 2022. “Role of RAS Signaling in Ovarian Cancer.” F1000Res 11: 1253. 10.12688/f1000research.126337.1. PMC9669513 36451660

[cbdd70409-bib-0028] Villagomez, F. R. , J. Lang , D. Nunez‐Avellaneda , et al. 2025. “Claudin‐4 Stabilizes the Genome via Nuclear and Cell‐Cycle Remodeling to Support Ovarian Cancer Cell Survival.” Cancer Research Communications 5, no. 1: 39–53. 10.1158/2767-9764.Crc-24-0558. PMC11705808 39625235

[cbdd70409-bib-0029] Wang, F. , S. Zhang , R. Jeon , et al. 2018. “Interferon Gamma Induces Reversible Metabolic Reprogramming of M1 Macrophages to Sustain Cell Viability and Pro‐Inflammatory Activity.” eBioMedicine 30: 303–316. 10.1016/j.ebiom.2018.02.009. PMC5953001 29463472

[cbdd70409-bib-0030] Wang, H. , J. Zhu , D. Zou , et al. 2025. “Multicenter Study of Ovarian Cancer Score for Diagnosing Ovarian Cancer.” Gynecologic Oncology 193: 58–64. 10.1016/j.ygyno.2024.12.017. 39793443

[cbdd70409-bib-0031] Wang, Y. , L. Zhao , J. Y. Luo , et al. 2025. “Nanomedicine‐Mediated Co‐Expression of a PD‐1 Blockade Agent and CXCL9 Synergizes T Cell Infiltration and Activation in Solid Tumors.” Biomaterials Science 13, no. 22: 6410–6422. 10.1039/d5bm01105a. 41054821

[cbdd70409-bib-0032] Xu, J. , Y. Fang , K. Chen , et al. 2022. “Single‐Cell RNA Sequencing Reveals the Tissue Architecture in Human High‐Grade Serous Ovarian Cancer.” Clinical Cancer Research 28, no. 16: 3590–3602. 10.1158/1078-0432.Ccr-22-0296. PMC9662915 35675036

[cbdd70409-bib-0033] Xu, L. , X. Kong , B. Zhang , et al. 2026. “Interplay Between Poly(ADP‐Ribosyl)ation and Specific Inner Cellular Events That Suggest Combination Strategies for Overcoming PARP Inhibitor Resistance.” Pharmaceutics 18, no. 3: 355. 10.3390/pharmaceutics18030355. PMC13028996 41900841

[cbdd70409-bib-0034] Yi, J. , M. Wu , Z. Zheng , et al. 2023. “Integrated Analysis of DNA Methylome and Transcriptome Reveals SFRP1 and LIPG as Potential Drivers of Ovarian Cancer Metastasis.” Journal of Gynecologic Oncology 34, no. 6: e71. 10.3802/jgo.2023.34.e71. PMC10627750 37417299

[cbdd70409-bib-0035] Zhou, H. , W. Wang , H. Xu , et al. 2024. “Metabolic Reprograming Mediated by Tumor Cell‐Intrinsic Type I IFN Signaling Is Required for CD47‐SIRPα Blockade Efficacy.” Nature Communications 15, no. 1: 5759. 10.1038/s41467-024-50136-z. PMC11233683 38982116

[cbdd70409-bib-0036] Zhu, X. , Y. Han , Z. Fang , et al. 2013. “Progesterone Protects Ovarian Cancer Cells From Cisplatin‐Induced Inhibitory Effects Through Progesterone Receptor Membrane Component 1/2 as Well as AKT Signaling.” Oncology Reports 30, no. 5: 2488–2494. 10.3892/or.2013.2680. 23970345

