# Supplementary material for: Integrative Multi‐Omics and Experimental Validation Define RELB as an Immune‐Related Prognostic Driver in Ovarian Cancer
Source: Chem Biol Drug Des. 2026 Oct 13;108(4):e70409. doi: 10.1111/cbdd.70409 (PMC13661770; doi:10.1111/cbdd.70409)
Supplement: Supplementary file 1 [file CBDD-108-e70409-s001.docx]

**Supplementory Material**

**Figure S1 Correlation of drug sensitivity**

(A-E) Sensitivity analysis of key genes and chemotherapy drugs.

**Figure S2 Analysis of immune differences**

(A-E) Differences in immune cell content of key genes between high and low samples.

**Figure S3 Immune correlation analysis**

(A-E) Correlation between key gene immunity and epidemic cells.

**Figure S4 Relationship between key genes and immune factors**

(A-E) Correlation of key genes with chemokine, Immunoinhibitor, Immunostimulator, MHC and receptor.

**Figure S5 GSEA analysis of key genes**

(A-E) Key genes involved in the KEGG signaling pathway, as well as pathway regulation and involved genes.

**Figure S6 Annotation of cells**

(A) We divided the cells into 12 clusters by UMAP algorithm based on the significant components available in PCA.

(B) Cell annotation of 12 clusters, 12 clusters were annotated into 7 cell types, namely, T cells, Fibroblasts, Monocytes, Epithelial cells, Endothelial cells, B cells, SMC.

(C) Bubble plot of 7 types of cells and cell markers.

(D) The difference in the proportion of 7 types of cells in the two samples.

**Figure S7. Expression of single cells**

(A) Scatterplot of key gene expression profiles in single cells.

(B) Bubble map of key gene expression profiles in single cells. Blue indicates low expression and red indicates high expression.
